# Positive antiphospholipid antibodies: observation or treatment?

**DOI:** 10.1007/s11239-023-02834-6

**Published:** 2023-06-01

**Authors:** Bogna Grygiel-Górniak, Łukasz Mazurkiewicz

**Affiliations:** Department of Rheumatology, Rehabilitation and Internal Diseases, Poznan University of Medical Sciences, Fredry 10, 61-701 Poznan, Poland

**Keywords:** Antiphospholipid antibodies, Rheumatic and non-rheumatic diseases, APLAs in a healthy population, Molecular mechanisms, Treatment

## Abstract

Antiphospholipid antibodies (APLAs) are  primarily directed toward phospholipid-binding proteins and are responsible for thrombotic events. APLAs include anti-β2Glycoprotein I (anti-β2GPI), anticardiolipin (anti-CL) antibodies, and lupus anticoagulant. These antibodies are typical markers of antiphospholipid syndrome (APS) and are a part of its diagnostic criteria. Many data underline the presence of APLAs in other rheumatic diseases (e.g., systemic lupus erythematosus, systemic sclerosis, Sjögren’s syndrome, rheumatoid arthritis and Behçet’s disease). However, they are also detected in patients with cancer, infection, and neurological disorders. Furthermore, healthy individuals may be carriers of APLAs. Chronic asymptomatic APLAs presence is most common in the elderly and subjects with chronic diseases (including malignancies). Specific kinds of APLAs are considered markers of oncological progression. These antibodies occur in 6% of pregnant women (without diagnosed APS) and are related to many pregnancy complications. Of worth, various types of APLAs are reported to have different prothrombotic properties. The risk of thrombotic events in APLA-positive but clinically naïve patients raises many questions in clinical practice. This manuscript analyses various clinical situations and consequences of the APLAs’ presence, particularly in patients without diagnosed APS. The prevalence, etiology, molecular background, and prothrombotic properties of numerous APLAs are broadly discussed. The new management approach in different clinical conditions and organ complications is present in the context of recent recommendations. Discussed data underlines that adequate and timely introduced thromboprophylaxis can decrease the risk of thrombus formation and prevent increased morbidity.

## Key points

APLAs are detected in APS; however, we can also find them in healthy individuals.

Cancers, obstetric complications, and infectious and rheumatic diseases coexist with APLA.

High-risk profile carriers require primary and secondary prevention.

The prevention depends on the presence or absence of documented thrombotic episodes.

Treatment recommendations depend on the type and severity of the pro-coagulative condition, but VKA or DOACs should be considered.

## Introduction

Antiphospholipid antibodies are involved in the pathogenesis of vascular and obstetric complications, prompting thrombotic states and inflammatory processes [1, 2]. The presence of APLAs is not only detected in antiphospholipid syndrome [2], but they are also found in a small percentage of healthy individuals, often discovered unintentionally [3, 4]. These antibodies do not cause thrombotic complications in healthy subjects because, according to the ‘second hit hypothesis,‘ some other factors can trigger clinical coagulation processes. For example, biological stressors (e.g., infections) can increase the risk of thrombosis in APLA-positive “healthy” subjects [5–7].

APLAs are necessary to diagnose APS; however, they have to coexist with thrombotic symptoms. Nevertheless, APS may develop in rare cases without fulfilling the diagnostic criteria [8, 9]. As a consequence, the prevalence of APS is challenging to estimate. Recent epidemiological studies show that the APS incidence in the American population is 2.1 per 100.000, and the prevalence—is 50 per 100.000 [8, 10]. In Europe (north-western Italy), the APS incidence is 1.1 per 100.000 with a prevalence of 16.8 per 100.000 [11]. Significantly lower prevalence is observed in Asia; for example, South Korea’s incidence achieves 0.75 per 100.000, and an APS prevalence is 6.19 per 100.000 [12]. Observed ethnic variations can be caused by environmental and genetic factors [13]. Though, during pregnancy and in patients with multiple co-morbidities, the APLAs prevalence increases dramatically. Andreoli et al. report that APLAs are detected in 6% of pregnant women, 13.5% of patients with stroke, and 9.5% with deep vein thrombosis [14].

Epidemiological data show that the mortality in patients with APS or positive APLAs is significantly higher than in the general population [8]. Thus, the detection and adequate diagnostic and clinical management is needed to prevent thrombotic complications. The “prothrombotic activity” analysis of various APLAs in clinically naïve patients is also necessary to assess the risk of possible complications. The presence of one kind of APLAs raises a question in clinical practice about its potential prothrombotic activity. This review analyses various clinical situations and consequences of the APLAs presence, particularly in patients without diagnosed APS. The broad approach to different practical problems is discussed, showing the possible scenario in the context of accessible data.

## Methods

**Fig. 1:**
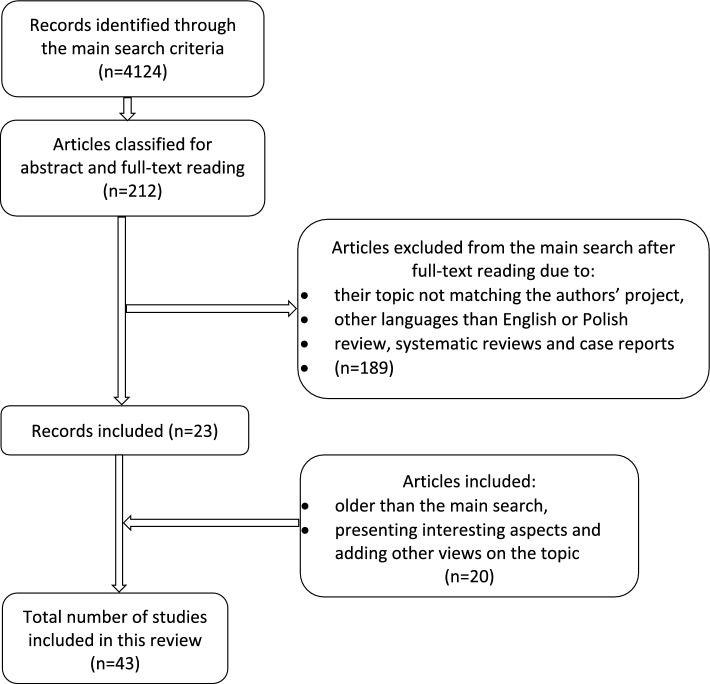
The graph presenting literature search methods used in this review

This review is based on a PubMed literature search involving various article categories, with an emphasis on original studies and meta-analyses. Case reports and reviews were excluded from the main search; however, they were used to discuss various aspects. Manuscripts were included by searching the words: antiphospholipid antibodies, antiphospholipid syndrome, anticardiolipin antibodies, lupus anticoagulant (LA), anti-β2Glycoprotein-I antibodies, malignancies, cancer, lymphoma, non-Hodgkin’s lymphoma, non-small cell lung cancer, pregnancy, asymptomatic carriers, infection, prevalence, clinical symptoms, and treatment. Conjunction words, like AND and OR, were used to define the search. The criteria of analyzed manuscripts included the time criterium and comprised articles published after 2011. Though to achieve the authors’ goals in this review and to present crucial data, some papers that did not match the inclusion criteria were allowed for this review. The authors’ search criteria are shown in Fig. 1.

### Molecular mechanisms of APLAs synthesis and activity

Various types of APLAs can be present in the specific constellation (co-existence with different kinds of antibodies) and related to clinical-specific complications. One of the APLAs is lupus anticoagulant (LA)—a heterogeneous group of immunoglobulins that act as acquired coagulation inhibitors by prolonging phospholipid-dependent in-vitro coagulation [15, 16]. LA binds to phospholipids and limits the possibility of attaching the prothrombinase complex, a crucial stage between coagulation and anticoagulation [17]. Prothrombinase activates thrombin, which participates in coagulation (leading to fibrin formation) in vivo and anticoagulation in vitro (Fig. 1). The reaction in vitro allows thrombin interaction with thrombomodulin causing activation of protein C [17]. Activated protein C (APC) inhibits factors V, VIII, and plasminogen activator inhibitor (PAI-1), causing a hypocoagulable and hyperfibrinolytic state [18, 19]. As a result, the APTT is prolonged in the presence of LA. Surprisingly, LA-positive patients are at higher risk of thrombosis (not bleeding observed in vitro) [20, 21]. This antibody is associated with an increased risk of deep vein thrombosis and pregnancy complications [22]. Thus, even causing prolongation of clotting times in vitro, LA paradoxically is associated with a pronounced tendency to thrombosis [23]. Unfortunately, this paradox’s exact background has not yet been explained [17, 21, 22].

**Fig. 2:**
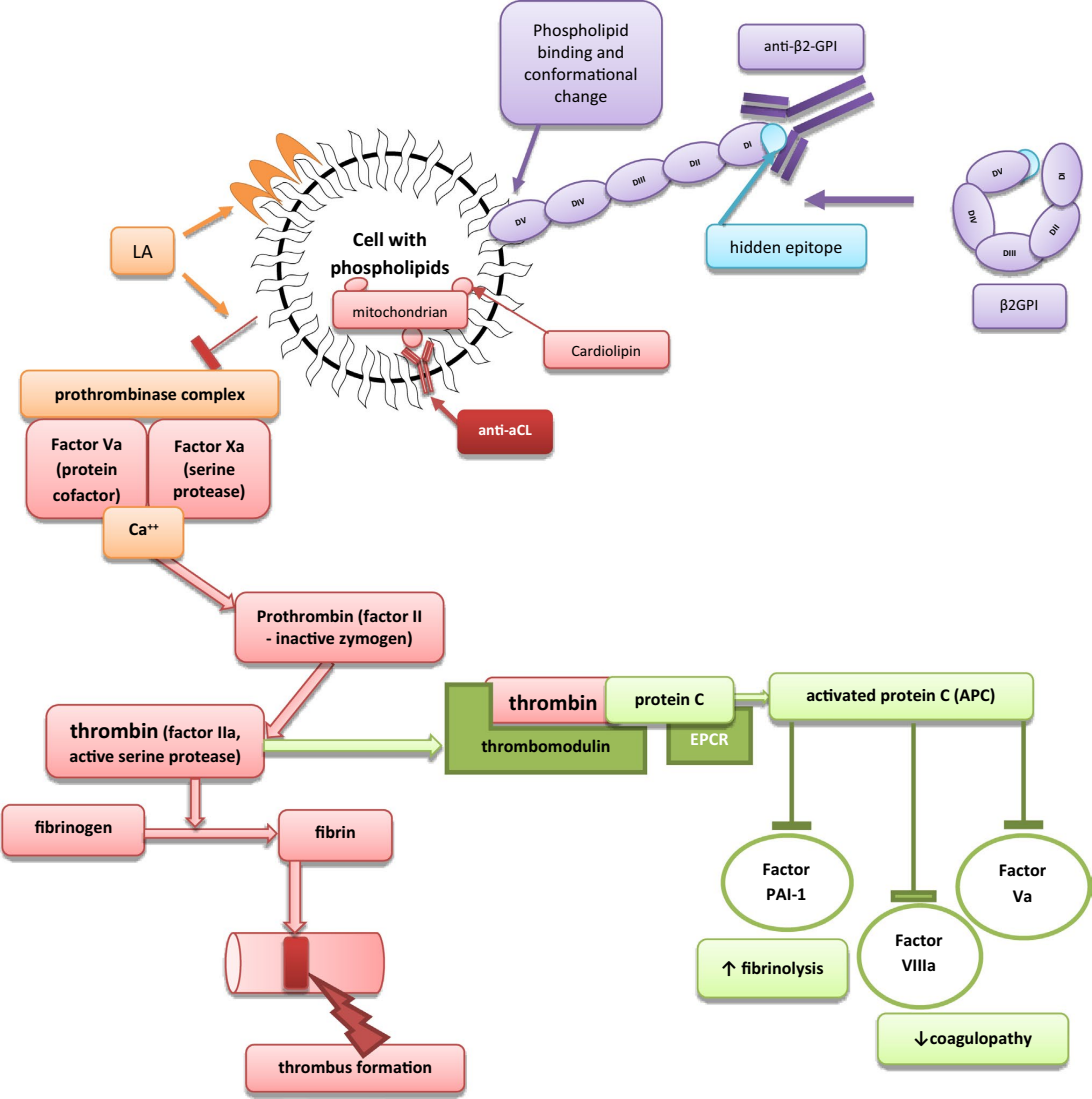
The pathological background of APLAs activity. Lupus anticoagulant (LA) binds to phospholipids and does not enable attaching the prothrombinase complex to the cell. The prothrombinase complex (factor Xa and Va) assembles on negatively charged phospholipid membranes in the presence of calcium ions. The prothrombinase complex catalyzes the conversion of prothrombin to thrombin. The enzyme thrombin has procoagulant activity because it converts fibrinogen to fibrin; however, if it binds to thrombomodulin and the endothelial protein C receptor (EPCR), it reveals anticoagulant properties by activating protein C (APC). APC cleaves activated cofactors Va, VIIIa, and plasminogen activator inhibitor (PAI-1), causing a hypocoagulable and hyperfibrinolytic state. Thus, LA is a class of APLAs, which causes a phospholipid-dependent prolongation of the clotting time but is associated with an increased risk of thrombosis and pregnancy complications. Beta 2—glycoprotein I (β2-GPI) consist in five domains (I–V), which can be present in two forms: open (J-shaped) and closed (circular). Domain V (DV) binds phospholipid, and its post-translational modifications cause a conformational change from the circular (closed) form to the open configuration. DV is responsible for binding to cell membranes. The open configuration causes the exposition of a previously hidden domain I (DI) epitope, which becomes a place of antibody binding. Anticardiolipin antibodies (anti-aCL) bind to cardiolipin on the mitochondrial surface and stimulate inflammation. Exacerbated inflammatory processes activate coagulation cascade and thrombosis

Another important antigen responsible for thrombotic-antithrombotic homeostasis is β2-Glycoprotein-I (β2GPI) (Fig. 2). It consists of five domains (I–V) β2-GPI and two conformational forms: open (J-shaped) and closed (circular) [24]. β2-GPI is a plasma protein of antithrombotic activity, which prevents protein S’s inhibition [20]. Typically, β2-GPI prevents von Willebrand factor (vWF) from binding with platelets, thus preventing aggregation. This mechanism is dysregulated in APS [25]. In this syndrome, interactions with anionic surfaces make conformational changes to the β2-GPI, followed by the exposition of a previously hidden epitope in domain I (DI) (Fig. 2). This epitope is a possible inducing factor in anti-β2-GPI antibody synthesis, whereas domain V (DV) is responsible for binding to cell membranes [26–28]. As a result, the concentration of antithrombotic protein β2-GPI decreases, which is considered one of the most crucial mechanisms in APS development. Subsequently, the formed β2-GPI-antibody complex binds to cellular receptors, increases pro-inflammatory activity, and stimulates various plasma cells, which participate in prothrombotic activation [20, 26]. Some factors (e.g., reactive oxygen species-ROS) may alter the configuration of β2-GPI to a dimeric form that strongly enhances antibody affinity [29].

The third antigen important in hemostasis is cardiolipin (diphosphatidylglycerol) (Fig. 2). It is a phospholipid located on the inner mitochondrial membrane [20, 30]. Cardiolipin is a crucial factor responsible for correctly assembling the mitochondrial respiratory super-complexes and other proteins involved in mitochondrial energy metabolism. It modulates the production of energy and participates in inflammatory processes [31]. The anticardiolipin antibodies (anti-CL) are not directed straight against anionic phospholipids (cardiolipin) but against proteins that create complexes with cardiolipin. One of the proteins is β2-GPI [32–34]. Single anti-β2-GPI and its complexes with cardiolipin are suggested to interact with endothelial cells and monocytes and induce a tissue factor-dependent procoagulant state [35]. Medium and high titers of IgM and IgG anti-CL antibodies are included in the Sapporo diagnostic criteria (APS classification criteria) [32, 36]. As a result, various thrombotic disorders and thrombocytopenia of the arterial and venous systems are observed [37].

### APLAs and cancer

APLAs are present not only in rheumatic diseases but also in multiple types of malignancies (Table 1). The various mechanisms of cancerogenic processes associated with a procoagulant state are suspected; however, the etiology of co-exitance APLAs with neoplasm remains not entirely unexplained [38]. Elevated APLAs are detected in hematologic malignancies such as solid cancers and lymphomas [39, 40]. Bairey et al. showed that an increased APLAs titer in non-Hodgkin lymphoma was a bad prognostic factor for general and event-free survival [41]. APLAs also contribute to the predictive value of the International Prognostic Index (IPI) [41]. For example, in lung adenocarcinoma patients, LA activity correlates with lower survival rates [42]. Therefore some authors suggest using laboratory tests detecting APLAs as a diagnostic tool, which may predict oncological outcomes [39].

**Table 1:** The most common APLAs in oncological patients

The most common APLAs in oncological patients			
Authors	Year	Malignancy type	Examples of APLAs
Bairey et al. [41]	2006	Non-Hodgkin lymphomas (NHL)	anti-β2GPI (IgG, IgM, IgA) anti-CL (IgG, IgM)
Vassalo et al. [40]	2014	Solid and hematological malignancies	LA anti-β2GPI (IgG, IgM, IgA) anti-CL (IgG, IgM)
Zhou et al. [45]	2011	Lymphomas	anti-β2GPI (IgG, IgM, IgA) anti-CL (IgG, IgM, IgA)
Zuckerman et al. [46]	1995	Various solid and hematological malignancies (mostly colorectal carcinoma, lung carcinoma and NHL)	anti-CL (IgG, IgM)
Miesbach et al. [47]	2006	Various solid and hematological malignancies	anti-CL (IgG, IgM) LA
Yoon et al. [39]	2003	Various cancer types e.g. NSCLC, colorectal cancer, ovarian cancer, breast cancer, and more	LA anti-CL (IgG, IgM) anti-β2GPI (IgG, IgM, IgA)
Shaukat and Hughes [48]	1990	Adenocarcinoma of the lung	anti-CL*
Ediriwickrema and Zaheer [44]	2011	DLBCL - case report	LA*

*A single case report; The most prevalent antibodies are underlined

*β2GPI* β2Glycoprotein-I, *IgM* immunoglobulin M, *IgA* immunoglobulin A, *IgG* immunoglobulin G, *LA* lupus anticoagulant, *CL* cardiolipin, *NSCLC* non-small cell lung cancer, *DLBCL* diffuse large B-cell lymphoma, *NHL* non-Hodgkin’s lymphoma

APLAs are also found in various solid malignancies. An interesting meta-analysis of Abdel-Wahab et al. summarizes the co-existence of solid tumors with APLAs [43]. For example, patients with gastrointestinal (GI) cancer have a level of anti-CL nearly 5 times higher than healthy controls. APLAs-positive lymphoid malignancies are typically associated with elevated PTT, normal PT, minimal extranodal disease, and potential thrombotic complications. Further, treatment with Rituximab-CHOP chemotherapy (cyclophosphamide, doxorubicin, vincristine, and prednisolone) leads to excellent clinical response with tumor remission and normalization of PT and PTT. Genitourinary (GU) and lung cancers are also frequently associated with the anti-CL presence. Stimulatingly, no statistically significant relationship existed between LA or anti-β2-GPI and solid tumors.

Furthermore, specific APLAs are more prevalent in particular solid tumors. For example, anti-CL is detected most often in GI and GU cancer. In turn, anti-β2GPI are found mainly in breast and lung cancer, while LA is detected in lung cancer [43]. Interestingly, LA can also be present in lymphoma. Such association was described by Ediriwickrema, and Zaheer presented a case report. A diffuse large B-cell lymphoma (DLBCL) was associated with LA, elevated PTT, PT, and INR. Fortunately, the treatment with Rituximab-CHOP chemotherapy led to a clinical response with tumor remission and normalization of PT and PTT [44]. Routine assessment of APLA in patients with solid cancer types remains debatable (Table 3). Abdel-Wahab et al. in their mata-analysis, point out that the current state of knowledge is insufficient to start routinely assessing APLA in these conditions. However, if the first detection of APLAs is positive, there is a need to repeat APLAs assessment to obtain clinical significance. Furthermore, APLA should also be assessed in every case of oncologic patients presented with clots [43].

### APLAs in infectious diseases

APLAs can also appear in many infectious diseases, such as hepatitis C-type (HCV) or Ebstein-Barr Virus (EBV) [49–51]. The occurrence of APLAs in viral diseases is associated with thrombotic events, including renal thrombotic microangiopathies or deep vein thrombosis [50]. Another viral infection accompanied by a hypercoagulability state is severe acute respiratory syndrome coronavirus 2 (SARS-CoV-2). Pneumonia, often associated with life-threatening respiratory complications, multiple-organ disorders, and thrombotic symptoms is the most severe SARS‐CoV-2 complication [52, 53]. The coincidence of COVID-19 with various thrombotic events is not fully understood yet [54]. A large meta-analysis investigating the prevalence of APLAs in SARS-CoV-2 infected patients revealed that APLAs were found in nearly 50% of the cases, and LA was the most frequently detected antibody [54]. Other authors reported similar prevalence and confirmed that LA was more commonly observed in COVID-19 patients than in other viral infections [55]. However, no correlation between the APLAs titers in COVID-19 and the risk of thrombosis was observed [54]. Conversely to viral infection, the co-occurrence of bacterial diseases and APLAs is less often associated with thrombotic events [49].

Another viral factor associated with APLA synthesis is HIV infection. APLAs stimulation in this opportunistic infection causes thrombotic complications, usually in the central nervous system (CNS) at an early stage of viral contamination. The single-photon emission computed tomography (SPECT) confirmed that brain perfusion abnormalities are often detected at the HIV onset [56].

APLAs synthesis is also observed in Q fever. This infection is characterized by fever and pneumonia caused by the intracellular *Coxiella burnetii* [57]. Anti-CL in class IgG is synthesized in the primary infection and incidentally associated with endocarditis development [57, 58]. Anti-CL antibodies are also present in arterial and venous thrombosis, particularly in severe courses of Q fever [57]. The diagnosis of Q fever-related APS can be problematic, mainly because this infection can mimic other conditions and can coexist with cholecystitis [59]. APLAs-positive Q fever associated with splenic infarction was also observed in children [60].

Interesting observations have been described by Gustafsson et al., who noticed elevated APLAs titters in SLE-smoking patients—mainly LA, anti-CL in class IgG, and anti-β2GPI in class IgG [61]. Augmented anti-CL and anti-β2GPI levels are also observed in SLE patients with periodontal bacteria colonization (*Porphyromonas gingivalis* and *Treponema denticola*) [62]. Smoking is also considered a crucial risk factor for periodontitis in the general population and can predispose to antibody synthesis—aCL (IgG and IgM) [63]. Interestingly elevated anti-CL in smokers with severe periodontitis makes them more prone to coronary heart disease [63].

Critically ill patients may also synthesize APLAs, yet their significance and role are not thoroughly described [64]. Likewise, in oncological malignancies, APLAs are considered a marker that may indicate mortality in critically ill patients with sepsis [65].

The data on the APLA presence in infectious diseases is scarce; however, some authors indicate the need for APLA assessment in patients suspected of APS due to clinical image [51]. Routine evaluation of APLA in COVID-19 disease is currently not recommended (Table 3) [54, 55].

### APLAs in the healthy population

APLAs are also detectable in healthy patients; however, only a small percentage of subjects with incidentally detected antibodies will develop antiphospholipid syndrome. Asymptomatic APLAs presence is most common in the elderly and patients with typical for their age chronic diseases (e.g., cardiovascular) [66]. For example, LA and hypertension are independent risk factors in asymptomatic APLAs carriers for a first thrombotic event [67]. Thus, in the elderly population is very difficult to define the risk of APS clearly. Some APLAs correlate with age, such as aCL or anti-β2GPI [66, 68]. A strong age-dependent increase in aCL and anti-β2GPI in class IgM was observed by Manukyan et al. [68]. Even though the literature discusses asymptomatic APLAs presence and possible risk factors for a first thrombotic event, many questions remain unanswered [66–68].

### The prothrombotic properties of APLAs

The co-existence of specific APLAs and clinical diseases predisposes particularly to thrombotic complications. For example, arterial hypertension and LA are crucial risk factors for thrombotic events in asymptomatic carriers [67]. In such cases, thromboprophylaxis should be considered at high-risk periods—pregnancy and immobilization [67]. As mentioned before, LA is a stronger risk factor for thrombosis than anti-CL [16]. Nevertheless, all types of APLAs associated with hypertension and hyperlipidemia increase the risk of thrombosis and miscarriages [69].

The impact of APLAs on the severity of thrombosis can be assessed by the antiphospholipid score (APL-S)—a tool for thrombosis prediction and the risk of APS [70]. Another scale, the Global Antiphospholipid Syndrome Score (GAPSS), is a quantitive device for APS based on positive APLAs tests and conventional cardiovascular risk factors [69]. The Adjusted Global Antiphospholipid Syndrome Score (aGAPSS) is another tool used in clinical practice to stratify APS patients at risk of recurrent thrombosis [71, 72] (Table 2).

**Table 2:** Tools for thrombosis prediction used in clinical practice

Scales used for thrombosis risk assessment		
APL-S	GAPSS	aGAPSS
Antiphospholipid score	Global APS Score	Adjusted Global APS Score
-Includes five clotting assays and six enzyme-linked immunosorbent assays: -anti-CL (IgG and IgM) -anti-β2GPI (IgG and IgM), -aPS/PT (IgG and IgM) -Enables the calculation of relative risks using OR to approximate the results for each antibody -The upper limit for each APLAs is 20 -APL-S score ≥ 30 is an independent risk factor for thrombosis [70]	-Scored based on calculations using multivariate logistic regression analysis -It includes six independent risk factors for thrombosis or pregnancy loss – hyperlipidemia, hypertension, LA, anti-CL (IgG and IgM), anti-β2GPI (IgG and IgM, and aPS/PT (IgG and IgM) -Points are given for variables proportionally to their regression coefficient [69]	-The scale includes the same criteria as GAPSS except for aPS/PT in class IgG and IgM (aPS/PT are not routinely tested) [71]

*APS* antiphospholipid syndrom, *OR* odds ratio, *aPS/PT* phosphatidylserine-dependent antiprothrombin antibodies, *anti-β2GPI* anti-β2Glycoprotein-I antibodies, *IgM* immunoglobulin M, *IgG* immunoglobulin G, *anti-CL* anticardiolipin antibodies

The 2019 EULAR recommendations on the management of APS in adults underline the existence of three risk profiles. Low-risk profile assumes anti-CL or anti-β2GPI at low-medium titers, also present transiently [1]. The medium-high risk profile is characterized by anti-CL in class IgG or IgM in higher titers (> 40 IgG/IgM phospholipid units, or > 99th percentile) or anti-β2GPI in class IgG or IgM in titer > 99th percentile [1, 36]. The high-risk profile is described by the persistent high titer of APLAs or LA present twice within a 12-week gap, or double-positive APLAs profile (any anti-CL, anti-β2GPI, LA combination) or triple-positive (all of mentioned APLAs types) [1]. Asymptomatic APLAs carriers with a high-risk profile should be treated with low-dose aspirin [1, 73].

### The prevalence of APLAs in rheumatic diseases

The typical rheumatic disease associated with detectable APLAs is an antiphospholipid syndrome. APS is characterized by thrombotic events such as venous or arterial thromboses, miscarriages, fetal deaths, or premature births [1, 36, 66, 74, 75]. The symptoms vary depending on the affected organ. Deep vein thrombosis is mainly observed in the lower extremities, and arterial thrombosis is the leading cause of cerebrovascular accidents like stroke or transient ischemic attack (TIA) [74]. APS can coexist with other autoimmune diseases, infections, and malignancies or can be classified alone without any previous definable conditions as primary APS [4, 66]. Secondary APS coexists with other disorders, mainly systemic lupus erythematosus (SLE) [74].

**Fig. 3:**
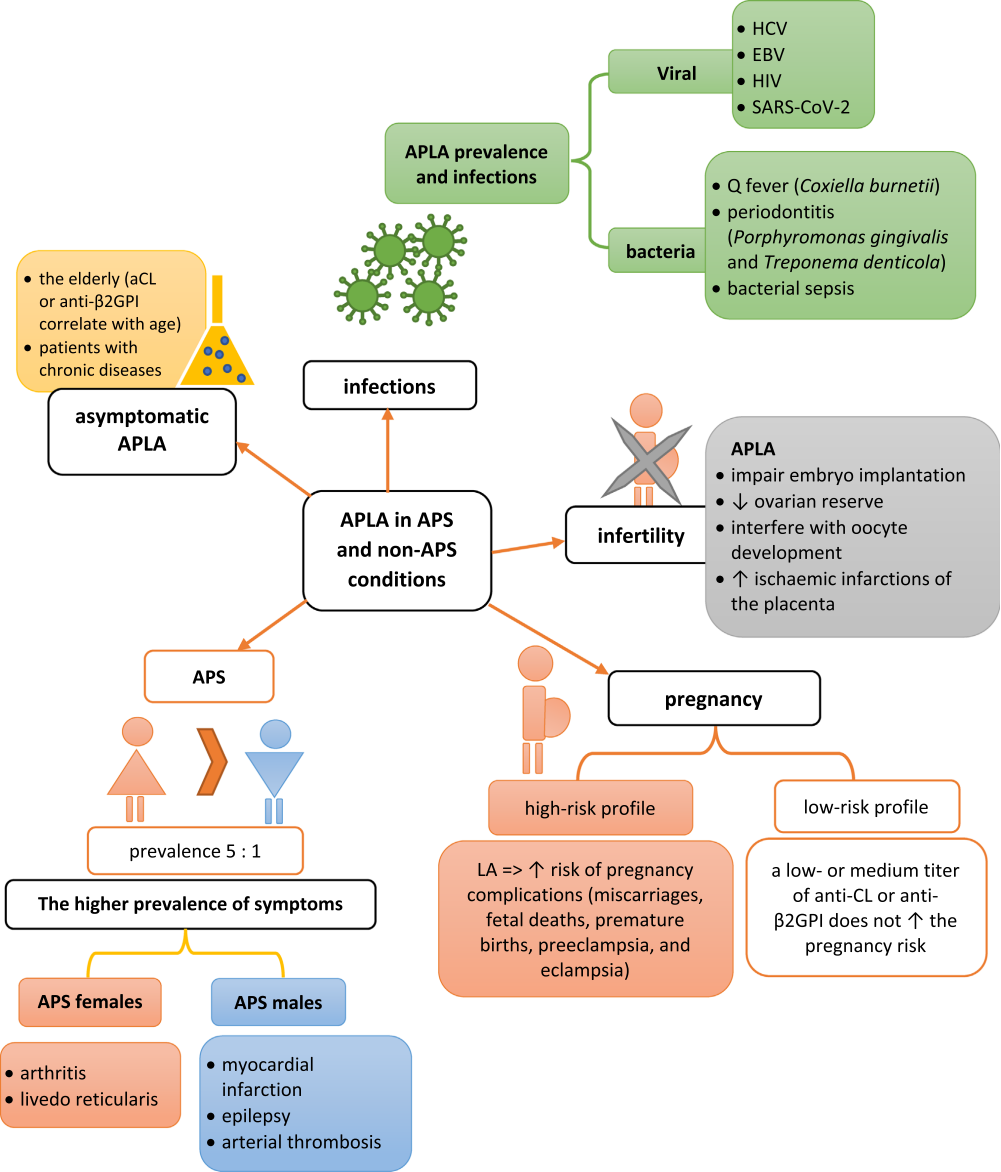
The prevalence and gender-related characteristics of APS

The epidemiological data show that women suffer from primary or secondary APS more often than men, with a female-to-male ratio is 5:1 (Fig. 3). Gender also seems to influence the presence of specific symptoms. For example, arthritis and livedo reticularis in APS are observed more frequently in females, whereas males suffer more often from myocardial infarction, epilepsy, and arterial thrombosis [76]. Additionally, SLE patients (both females and males) with coexisted APS have a much higher risk of organ damage and death than subjects with SLE alone [77].

APLAs are detected not only in APS but also in other connective tissue diseases such as systemic sclerosis (SSc), Sjögren’s syndrome (SS), rheumatoid arthritis (RA), and mixed or undifferentiated connective tissue disease (MCTD) [78]. The median prevalence of APLAs in CTD is about 14.05% of patients, most common in SSc subjects among mentioned above diseases [78]. In SSc, endothelial damage is observed, leading to vascular changes in many tissues and organs [79]. Unfortunately, APLAs contribute to vascular abnormalities present in SSc [80]. Merashli et al. described anti-CL and anti-β2GPI, which increased the prevalence of SSc complications (pulmonary arterial hypertension, renal disease, thrombosis, and digital infarction) [79].

Primary Sjögren’s syndrome (pSS) is mainly associated with LA, which is a predictor of stroke and deep vein thrombosis [81]. The prevalence of APLAs in RA is about 28% [82]. When aCL is present, patients suffering from RA tend to have a higher disease activity and more often develop extra-articular findings. The mechanism of this phenomenon is yet unknown [83]. Furthermore, APLAs are a poor prognostic factor in RA [83].

APLAs are also detectable in some vasculitis, e.g., Behçet’s disease (BD). This vessel inflammation is characterized by mucosal and skin ulcerations in populations with genetic predisposition [84, 85]. The most prevalent APLAs in BD are anti-CL and anti-β2-GPI, which are responsible for developing vascular pathologies, such as arterial and venous thrombosis [86].

In rheumatic and musculoskeletal diseases with positive APLA, routine screening for the antibodies is not supported enough by the literature; however, in some conditions like pregnancy planning or thromboembolic events in a patient’s history, assessing APLA might be beneficial [78]. Pasoto et al. gravitates towards the appropriateness of LA detection in pSS, as LA is an important marker for APS and stroke (Table 3) [81].

**Table 3:** The need for APLA testing and management with enough literature support

The need for APLA testing and management with enough literature support		
Condition/group of conditions	APLA testing according to literature	Treatment
Solid tumors	Not routinely [43]	
Infectious diseases	Not routinely [51]	
Rheumatic and musculoskeletal diseases	Not routinely [78]	
Solid and hematological malignancies	NO [40]	
Covid-19	NO [54, 55]	
pSS	YES-LA [81]	
Asymptomatic APLA carriers—high risk profiles		Primary prophylaxis - low dose aspirin [1, 101]
APS (with documented thrombosis episodes)		Secondary prophylaxis: 1) VKA therapeutic INR target 2–3 or DOAC if contraindications (NOT rivaroxaban in triple APLA positive) [1] or 2) DOAC-single or double APLA positive patients [101] VKA-triple APLA positive patients [101]
APLA positive women/OAPS		1) LDA before conception - In high-risk profiles with no previous history of thromboembolism or obstetrical complications [1] 2) LMWH (+ LDA) if miscarriages previously [1, 97] VKA are contraindicated! The published data underline the positive influence of hydroxychloroquine or prednisone, which can be used safely and successfully during pregnancy [1, 104, 105].

### APLAs prevalence in pregnancy and infertility

Women without diagnosed APS but with high positive APLAs during pregnancy have an increased risk of unfavorable pregnancy outcomes [87]. Low-risk APLAs profile assumes the occurrence of a low- or medium titer of anti-CL or anti-β2GPI [2, 75]. The presence of LA is associated with major pregnancy complications. The study of Lockshin et al. confirmed this statement and showed that the absence of LA, while single anti-CL or anti-β2GPI are present, does not increase the pregnancy risk [88]. Recently published analysis shows a correlation between LA’s positivity and a shorter gestation period (37.1 vs. 38.5 weeks), which is strictly connected to pregnancy risk [89].

Epidemiological studies revealed that APLAs are more often positive in infertile women [90]. The precise mechanisms of primary infertility are unknown, but some hypotheses are trying to explain APLAs’ influence. Various data underline that APLAs can cause interference with endometrial decidualization (which leads to embryo implantation impairments), decrease ovarian reserve, and interfere with oocyte development and ischaemic infarctions of the placenta [91–94]. Yamakami et al. show that ovarian reserve may be decreased in more than 50% APS women [94].

Assisted reproductive treatment (ART) techniques are widely used to treat various infertility forms [95]. A meta-analysis of ART effectiveness revealed that in-vitro fertilization (IVF) or intracytoplasmic sperm injection (ICSI) brings a higher miscarriage rate in APLAs-positive women than APLAs-negative; however, the live-birth rates are comparable [95]. Thus, the APLAs measuring is suggested in every woman before ART to predict outcomes and facilitate early intervention [95]. Repeated unsuccessful ICSI or IVF is called recurrent implantation failure (RIF). Unfortunately, the most frequent RIF is described in APLAs-positive women, particularly with positive anti-CL in class IgG [96].

### Pregnant women with APS

Obstetrical APS (OAPS) is characterized by miscarriages, fetal deaths, or premature births [1, 2, 66]. In some cases, intrauterine growth restrictions are also observed [97]. Preeclampsia and eclampsia are among the most threatening conditions [97]. Unfortunately, the conventional treatment of women with OAPS does not guarantee an uneventful pregnancy and the delivery of healthy neonates [98]. The clinical risk of fatal obstetric outcomes is often associated with a high LA level [2, 88, 99]. Thus, Latino et al. suggest analyzing the APLAs profiles in every woman with obstetric APS, which enables the evaluation of pregnancy risk and allows to monitor of the potential benefits of antithrombotic treatment, which gives the best effect if started in the first trimester [98].

### A recommendation for APLAs-positive carriers

Patients with positive APLAs require primary and secondary thromboprophylaxis [1, 100]. Primary prophylaxis should be concerned in asymptomatic APLAs carriers, especially with-high risk profiles (previously defined in this manuscript). Recommendations suggest treatment with low-dose aspirin (LDA) [1, 101]. In patients with APS (with documented thrombosis episodes), secondary thromboprophylaxis should be treated with anticoagulative medications [102]. However, the treatment recommendations differ between published guidelines. The first-choice therapy indicated by EULAR is the vitamin K antagonist (VKA), which should be adequately dosed to allow for achieving a prophylactic INR target of 2–3 [1]. VKA can be replaced with Direct Oral Anticoagulants (DOAC) if there are contraindications for VKA use, or DOACs could be added to VKA treatment if targeted INR is not achieved [1]. The International Congress on Antiphospholipid Antibodies points out the need to consider DOACs because they are the first-choice therapy in patients with the first venous thrombotic event in the general population [101]. DOACs can be considered in single or double APLA-positive subjects. However, VKAs are recommended in triple APLA-positive patients [101]. Of worth, rivaroxaban, according to EULAR recommendations, should not be used in triple-positive patients because this medication use was associated with the risk of recurrent thrombosis [1].

As mentioned, obstetric APS (OAPS) describes obstetrical pathology problems related to APLAs [97]. Women diagnosed with APS do not have contraindications for pregnancy, but a multidisciplinary team should control the pregnancy to prevent thrombotic complications [97, 103]. It means that pre-conception prevention is very important [103]. In high-risk profiles with no previous history of thromboembolism or obstetrical complications, LDA before conception should be considered [1]. Low molecular weight heparin (LMWH) is recommended after the pregnancy confirmation in addition to previous LDA treatment if miscarriages were observed previously [1, 97]. Vitamin K antagonists should be replaced with low molecular weight heparin (LMWH) and LDA during pregnancy. VKA can be introduced once again in postpartum [97]. LMWH subcutaneous injections should be used up to 6 weeks after delivery [1, 97]. Some studies suggest using additional anti-inflammatory drugs if recurrent abortions were observed previously during APS. The published data underline the positive influence of hydroxychloroquine or prednisone, which can be used safely and successfully during pregnancy [1, 104, 105].

**Fig. 4:**
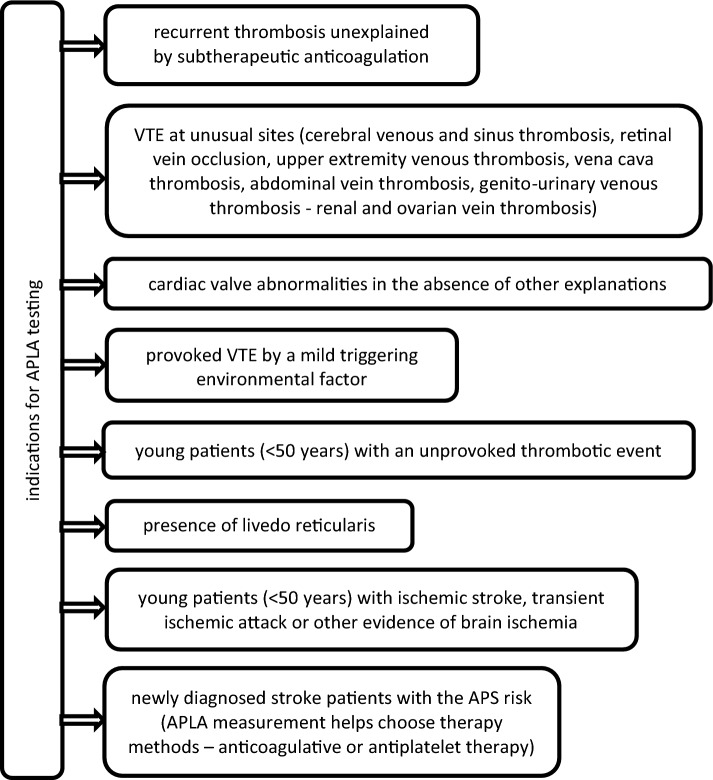
Indications for APLA measurement. *VTE* venous thromboembolism

Besides mention above conditions, APLA measurement should be done in every patient with diagnosed or suspected thrombotic complications. According to guidelines of the British Society for Haematology (BSH) and ISTH (International Society on Thrombosis and Haemostasis), antiphospholipid antibody testing should be performed when there are clinical features suggestive of APS [102, 106–108]. The presence of APLA and raised D-dimer levels are independent risk factors for recurrence after a first unprovoked VTE [109]. The indication for APLA testing is shown in Fig. 4.

## Conclusion

Though assessing APLAs positivity in the populations is complex, genetic and environmental factors play a role in their development. Vascular and obstetric complications are serious problems resulting from APLAs positivity. Accidental APLAs detection rarely triggers thrombotic events. These antibodies are found in a healthy population and coexist with various clinical conditions. Besides rheumatic diseases, they are observed in malignancies, infections, and epilepsy. APLAs can cause multiple pregnancy complications in APLAs-positive women or women with diagnosed OAPS. Adequate and timely introduced thromboprophylaxis can decrease the risk of thrombus formation and prevent increased morbidity of mother and fetus.
